# Stigma associated with cutaneous leishmaniasis in rural Sri Lanka: development of a conceptual framework

**DOI:** 10.1093/inthealth/ihae021

**Published:** 2024-03-15

**Authors:** Hasara Nuwangi, Lisa Dikomitis, Kosala G Weerakoon, Chandani Liyanage, Thilini C Agampodi, Suneth B Agampodi

**Affiliations:** Department of Community Medicine, Faculty of Medicine and Allied Sciences, Rajarata University of Sri Lanka, Saliyapura, Sri Lanka; Centre for Health Services Studies and Kent and Medway Medical School, University of Kent, Canterbury, UK; Department of Parasitology, Faculty of Medicine and Allied Sciences, Rajarata University of Sri Lanka, Saliyapura, Sri Lanka; Department of Sociology, Faculty of Arts, University of Colombo, Colombo, Sri Lanka; Department of Community Medicine, Faculty of Medicine and Allied Sciences, Rajarata University of Sri Lanka, Saliyapura, Sri Lanka; International Vaccine Institute, Seoul, South Korea

**Keywords:** leishmaniasis, neglected tropical diseases, skin diseases, Sri Lanka, stigma

## Abstract

**Background:**

There is limited knowledge about the stigma associated with cutaneous leishmaniasis (CL) in Sri Lanka. To ensure that leishmaniasis researchers focus on CL-associated stigma, we provide an evidence-based framework that can be used in future research.

**Methods:**

We conducted a systematic review on CL-associated stigma using international evidence and carried out a multimethod qualitative study in the Anuradhapura district in Sri Lanka. Based on that, we identified manifestations of stigma, drivers and facilitators that we synthesised to develop a conceptual framework on CL-associated stigma.

**Results:**

Our framework consists of drivers, facilitators and self-stigma experienced by people with CL. Stigma drivers included fear, misbeliefs and misconceptions about CL; the belief that wounds are disfiguring; the treatment burden and implied blame. Facilitators that reduced stigma included knowledge of the curability of CL and awareness that CL is not contagious. The nature of social interactions in rural communities enhanced stigma formation. We identified various enacted, felt and internalised stigma experiences of people with CL.

**Conclusions:**

We developed a conceptual framework of the stigma associated with CL that can be used to develop targeted interventions to increase CL awareness, address stigma and improve the quality of life for CL patients.

## Introduction

Cutaneous leishmaniasis (CL) and mucocutaneous leishmaniasis (MCL) are stigmatising neglected tropical diseases (NTDs) due to the potential physical disfigurement they may cause.^1,2^ CL causes nodules or plaques in exposed areas of the skin that can develop into non-healing single or multiple ulcers.^3–5^ MCL manifests as lesions in the nasal and oral cavity, the subglottic region and trachea.^6,7^ Health-related stigma is the ‘social disqualification of individuals and populations who are identified with particular health problems’.^8^ Health-related stigma can also be associated with poor prognosis, increased risk of disability, delay in diagnosis and treatment and continuing risk of disease transmission. This can result in increased morbidity and psychological problems such as depression, stress and fear.^8–10^ Hence, understanding CL-associated stigma is important in preventing, treating and controlling the disease.

A number of frameworks have been used to describe health-related stigma. Bos et al.^11^ present a conceptual framework comprising four interconnected manifestations of stigma: public stigma, self-stigma, stigma by association and structural stigma. The health stigma and discrimination framework of Stangl et al.^12^ illustrates the various stigma experiences and practices, including lived realities, beliefs, attitudes and actions through which stigma manifests, as well as the drivers and facilitators of stigma. In this framework, Stangl et al.^12^ describe ‘drivers’ as inherently negative and ‘facilitators’ as either negative or positive. These drivers and facilitators of stigma vary according to the health condition. The occurrence of stigma ‘marking’, whereby a stigma is applied to individuals, is determined by both drivers and facilitators.^12^ However, there is poor assimilation of real-life contextual manifestations into theoretical frameworks in describing CL-associated stigma.^13^

In Sri Lanka, leishmaniasis has been recognised as a public health concern since 1904.^14^ The country's context is unique, as CL appears to be a re-emerging disease and awareness of CL remains low.^15^ CL was declared a notifiable disease and reintegrated into the public health surveillance system in 2008.^16,17^ Since then, there has been a notable increase in reported cases.^18^ However, there is limited research around CL-associated stigma in Sri Lanka.

In 2010, Fernando et al.^19^ reported that in 120 individuals, 18% of males and 25% of females experienced isolation and social stigma associated with CL. Among them, 17 patients reported negative reactions from their families due to their wounds. Studies report that CL wounds affect the quality of life of patients in Sri Lanka, but the underlying reasons were not explored.^20,21^ Our recent research shows a notable delay in healthcare seeking for CL,^22^ along with a significant psychosocial burden associated with CL in Sri Lanka.^23^ Given that CL is associated with poverty and other inequalities, early identification of the drivers, facilitators and experiences of CL-associated stigma to prevent ill mental health and quality of life of affected individuals is important.^24,25^ This evidence demonstrates a clear lack of focus on CL-associated stigma in Sri Lanka. To date, we lack a comprehensive conceptual framework pertaining to CL-associated stigma, as shown in our recent systematic literature review.^13^ Here, we rectify this gap by proposing a conceptual framework, based on our synthesised evidence for understanding CL-associated stigma, including manifestations, drivers and facilitators in the local context.

## Methods

**Figure 1.**
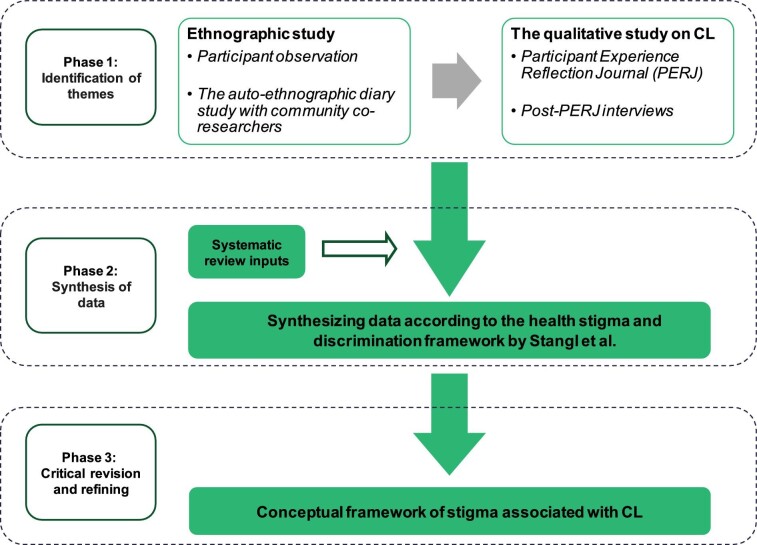
Steps in the development of the conceptual framework.

This study, conducted as part of the ECLIPSE program (Empowering people with cutaneous leishmaniasis: Intervention program to improve patient journey and reduce stigma via community education),^26^ utilised a multimethod qualitative design consisting of an ethnographic study and a qualitative study of people with CL in three selected communities with high CL prevalence in Anuradhapura, Sri Lanka.^23^ We developed a conceptual framework based on our systematic review^13^ and qualitative dataset (Figure 1).^11,23,27^

**Figure 2.**
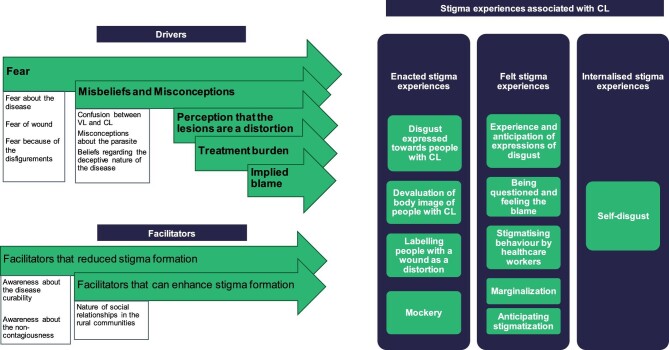
The conceptual framework of CL-associated stigma.

### Exploring the lived experiences of people with CL

From January 2021 to January 2022, we conducted ethnographic fieldwork in Anuradhapura, a district in Sri Lanka with a high prevalence of CL. Qualitative data were collected through participant observation: ECLIPSE researchers lived in the communities and an auto-ethnographic diary study was conducted with community co-researchers. In our ethnographic study, we explored the culture, social context and health beliefs and behaviours to understand the CL-associated stigma within the context. The ECLIPSE researchers conducted participant observation by engaging in daily communal life in the communities for 4 months. Because of COVID-19 pandemic restrictions, conventional fieldwork had to be halted. We were able to implement alternative culturally appropriate data collection methods that ensured community members were not exposed to COVID-19 risks.^28^

Because of the high literacy levels of the Sri Lankan rural communities,^29^ we were able to train community members (n=30) to document their daily routines and observations in diaries. This, in turn, was followed up with interviews conducted by researchers with the diary writers (n=26). This was followed by collecting participant experience reflection journals (PERJs) and post-PERJ interviews.^23,28^ PERJs were co-developed with the community using community engagement and involvement principles. It consisted of 11 open-ended questions on the lived experience of CL patients. Community members were involved in designing the questions, using context-appropriate terms and phrases. The detailed account of the PERJ development process is published elsewhere.^23,28^ The participants (CL patients) completed the PERJs (n=30) and we conducted post-PERJ interviews (n=25) to understand the documented experiences in depth. The few participants who had lower literacy levels (n=2) were given the opportunity to write the journal/diary with assistance from a trusted family member. The details of the participants are provided in the supplementary materials.

### Systematic review of the stigma associated with CL and MCL

As stigma in CL is poorly conceptualised at the global level, we conducted a systematic review to analyse and synthesise the stigma manifestations associated with CL and MCL.^11,13,27^ We identified the cognitive, affective and behavioural manifestations of public, enacted, felt and internalised stigma associated with CL and the tools used to measure stigma.

### Data analysis and development of the conceptual framework

The conceptual framework of stigma was developed in three phases: phase 1, identification of themes; phase 2, synthesis and resynthesis of data and phase 3, critical revision and refining of the framework by the research team.

#### Phase 1: identification of themes

Through thematic analysis of the qualitative data, we identified potential drivers and facilitators and experiences of stigma by analysing four datasets: ethnographic field notes, auto-ethnographic diaries, post-diary interview transcripts, PERJs and post-PERJ interview transcripts.^30^

#### Phase 2: synthesis and resynthesis of data

The systematic review outcomes^27^ informed our identification of different stigma manifestations and experiences. In this phase, we adopted the six steps Jabareen^31^ suggested in developing a conceptual framework to synthesise data. We used the health and discrimination framework of Stangl et al.^12^ to categorise and interpret drivers and facilitators of stigma identified in phase 1.

#### Phase 3: critical revision and refining of the framework by the research team

During this phase, the entire ECLIPSE team engaged in a critical review of the framework. We refined the descriptions of the themes and made necessary revisions to the framework.

## Results

### Drivers of stigma

#### Fear

Fear about CL, especially regarding the wound and the potential disfiguration, led to stigmatisation. The unfamiliarity with CL heightened anxieties and stories and misconceptions fuelled the uneasiness.

The slow healing process, the appearance of the wounds and the possibility of recurrence of wounds were concerns leading to fear. Some study participants compared the CL wounds to cancer, perceiving them as widespread and comparable to a malignant condition:

…you get wounds all over your bodies from sandfly's disease. It's like a cancer. (J19)

People with smaller wounds/papules/nodules also feared getting big visible wounds later. They mentioned that, in general, the wounds one gets from CL are frightening:

The wound in my finger looked scary. (J15)

#### The perception that the wounds are a disfigurement

The perception that the wounds caused disfigurement contributed to the manifestation of stigma and its perpetuation.

Some people have big wounds. It's a complete disfiguration. (J10)

People expressed non-verbal signs of disgust when talking about CL wounds, such as wrinkling their noses.

#### Misbeliefs and misconceptions about the nature of CL

Participants tend to have local beliefs and conceptions that lack plausibility according to the current biological or medical knowledge. We conceptualised these misbeliefs and misconceptions as drivers of stigma acting through the representation of the ‘perceived dangerousness of the disease’.^11^ People confused visceral leishmaniasis (VL) and CL, leading study participants to think that CL, just like VL, affects one's internal organs, including the kidneys, lungs and heart, and is potentially fatal. Another misconception was regarding the sandfly. Commonly, people believed that the sandfly lays eggs on the body and a worm emerges from these eggs and travels inside the body, causing internal organ damage. People perceived CL as a deceptive disease capable of causing harm without apparent symptoms. Their explanation was rooted in their farming experiences.

Just as the fruit fly damages the whole fruit from the inside, this disease damages your inside without showing any symptoms from outside. (J01)

The method of communication of health messages and the use of fear to promote treatment compliance played a role in fostering misconceptions.

The doctors told us when the sand fly bites, they lay eggs on us. They told us to take treatments properly because it can affect internal organs. (J07)

#### Treatment burden

The prolonged duration of treatment further reinforced the perception of the disease as perilous and different from other diseases.

It's not like other wounds. Some people have gone through treatments for years. Imagine how dangerous the disease is? (J21)

In addition, the regular clinic visits for treatment were a cause of stereotyping people with CL as ‘patients’.

#### Implied blame

There was also an inclination to blame individuals with large wounds for not seeking treatment earlier, relating to the representation of the controllability of the condition by an individual.^11^

I saw a person with a very large wound, I don't know why he didn't seek medicine before it got worse. If it was me, I would have probably taken medicine when it was very small. (J09)

### Facilitators that reduced stigma formation

#### Awareness about the curability and non-contagiousness of the disease

Learning that CL is curable helped people to cope with fear.

I was afraid at first but later learned that this disease is curable, and I met a few people who were cured as well. This reduced my fear. (J11)

We also observed this during ethnographic fieldwork; when community members are knowledgeable of the curability, they tend to advise and help patients to seek medical care, reducing the occurrence of stigmatisation. The general public who are unaware of the disease did not recognise CL wounds to be contagious, as it resemble, a nodule or a boil due to an insect bite, which is common in agricultural communities. The CL patients and close contacts who knew about the disease knew that the disease is not contagious.

If it's contagious, we would distance ourselves from a person. But the sand fly's disease is not contagious like other skin diseases (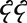-*dhada*  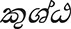-*kushta*). (J29)

In the interviews, the participants emphasised the importance of public awareness in reducing stigmatisation.

If people are aware that the disease is curable and not contagious, they will consider it another normal disease. They won't disgust anyone. There is a chance people will think it's contagious if they are not educated about the disease. (J19)

### Facilitators that can enhance stigma

The nature of social relationships in rural communities might facilitate stigmatisation. People routinely gather and gossip, for instance, at the village shop in the evening after work. People with CL tend to avoid such gatherings, deviating from their routine to avoid being subjected to questioning. Likewise, within the dermatology clinic setting, people regularly interacted with people with large wounds, which, coupled with prevailing myths regarding the perceived danger, severity and controllability of the disease, contributed to stigmatising attitudes.

### CL stigma manifestations and experiences

#### Public stigma

Although people with CL experience various self-stigma experiences (felt, enacted, internalised), there is currently little to no public stigma associated with the disease within these communities. During ethnographic fieldwork, we did not observe actual instances of people being stigmatised or discriminated against because of CL. The communities did not exhibit direct stigmatising cognition, affection or behaviours in their interactions or discussions about people with CL. The common consensus of people often did not support the stigmatisation of CL patients. We observed several reasons that could contribute to low levels of public stigma in CL in Sri Lanka. Our ethnographic study indicated that the general public is mostly unaware of the disease unless there are patients being diagnosed and treated in their neighbourhood. Second, the early clinical manifestations of CL resembled common non-harmful skin conditions such as insect bites, pimples and warts that do not cause curiosity among patients or family members.

Most people are not afraid of this disease simply because they are not familiar with it. Only a handful of people around here actually knows about this disease. Also, we haven't had anyone in our village suffer from a severe case [a large, visible CL wound], so people haven't really grasped how dangerous it could be. (J04)

Third, as described above, the facilitators that reduce stigma inhibit generation of public stigma. However, our analysis of the PERJ and PERJ-based interview data indicate that potential drivers of stigma exist among patients. The drivers we describe above could contribute to establishing public stigma unless prevention and control activities are not established at this initial stage of the re-emergence of the disease.

### Enacted stigma and intersecting stigmas

#### Disgust expressed towards people with CL

Study participants explained that they were disgusted by people with large visible wounds. Some found it difficult to articulate the intensity of their disgust, merely describing it as deeply repugnant.

I don't know how to explain that. All I can say is that it is very disgusting to look at. (J01)

During our ethnographic study, we discovered that community members and their families generally exhibited disgust and marginalisation towards individuals with all types of large, visible wounds (not limited to CL wounds). Hence the existing stigma for wounds intersected with the disease. One participant shared the experience when her father had a large non-CL wound.

Because of my father's wound, we actually faced a difficult situation. Others didn't like coming to our place. But I understand it, even we would not have even have a glass of water from a house where there is a person with a big, smelly wound. (J02)

Participants mentioned that people with CL wounds would have to cover the wounds to get by in day-to-day life and to avoid feeling shame.

Listen, this is not a nice thing to say. But the thing is, those wounds (CL wounds) are disgusting. So, if you have such a wound, it will be difficult for you to face society. You will have to keep the wound covered. (J07)

#### Perceptions on body image of people with CL

People with big wounds were often called ‘ugly’ (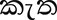-*ketha*), especially facial wounds. People talked about the scar impacting a person's beauty. They said a person with a visible scar would feel uncomfortable around others:

The issue is that the scar remains even if you are healed. There will be a black-coloured scar on your face where you will feel embarrassed. (J07)

#### Labelling people with a wound as disfigured

The term ‘disfigurement’ (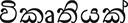-*wikruthiyak*) was frequently used to describe both CL wounds and the individuals affected.

If you get a wound on your face, then you become a disfigured person. (J01)

People believed that those with disfigurements might need to seek alternative employment, such as privately managed occupations like farming. The notion of disfiguration was associated with misconceptions as well.

I heard that the sandfly lay eggs on you, and that creature that comes from the eggs will make more wounds. And then you become more and more disfigured as you will get more and more wounds. (J10)

#### Mockery

Young girls with CL were subjected to mockery because of having a wound on the face and trying to cover it. Within Sri Lankan culture, people often use humour and playful teasing in their interpersonal interactions. However, being mocked because of CL creates distress and can lead to stigma, particularly regarding the intersecting gender concerns. A participant described an incident involving a young girl at the clinic. She had a facial wound that she kept covered with a cloth. A participant described how several young males in the clinic mocked her and laughed at her, saying,

Show us your face…why are you covering it up? (J21)

### Felt stigma experiences

#### Experience and anticipation of expressions of disgust

People with CL expressed disappointment with life after contracting the disease and perceived others’ gazes as conveying expressions of disgust.

I was disappointed with life after contracting this disease. If you don't take medicine at the right moment, you will have wounds all over your body like us. The others look at us with disgust. (J24)

We identified that people anticipating such gazes covered up their wounds to avoid negative reactions of the public.

People looked at my hand (with a wound) strangely. Later I used to cover up the wound. Even when I am going to work. I'd put some ointment and place a bandage over the wound. If the wound is visible, then others disgust it. (J19)

#### Being questioned and feeling the blame

People with CL reported feeling irritated by questions from others about the disease and the wound. Some people reported feeling disheartened by the questions, as they perceived being questioned as blaming them for contracting this disease.

People in my office kept asking me about the wound and why it is not yet healed. I felt disheartened and sad because I felt like they were accusing me of having contracted this disease. It's not like I asked for this to happen to me. (J05)

#### Stigmatising behaviour by healthcare workers

There were instances where healthcare workers’ actions caused people with CL to feel embarrassed and marginalised. A 65-year-old CL patient expressed his experience:

One time at the clinic, a minor staff called us hama…hama (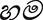 [the Sinhala word for skin]). Everybody else looked at us and laughed at us, imagine calling us that? It's one of the most degrading things to call a person (with skin disease). Even the gentlemen working in the medicine counters laughed at us. (J04)

It is interesting to note that sometimes the sympathy and attempts by doctors to assure people that the disease is not stigmatising left them feeling stigmatised.

We were taken inside a room, and a doctor talked to us. He said we should not worry, and nobody will marginalise or treat us differently. For them to say that, it should be true, right? People must be marginalising CL patients. That's how I felt when the doctors told me that. (J21)

#### Marginalisation

People with large visible wounds have shared their experiences of being marginalised (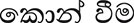-*kon weema*) with fellow CL patients at the clinic. According to the affected person, lack of awareness about the disease was the reason for marginalisation.

There was an old person at the clinic with a big wound on his leg. It was infected and pus-filled. He used to complain to me that he was marginalised by his own community. He said the villagers didn't like him coming to public places and meetings and was forcefully dismissed by people. He said people didn't know about this disease, and they feared it. (J02)

#### Anticipating stigmatisation

Some people shared they felt sad because they expected some level of marginalisation, and some people had limited engagement with others because they expected negative treatment. It was apparent that even in the absence of negative experiences, they anticipated that they would face such treatment and took preventive measures. People mentioned that they felt uneasy engaging with society. This was due to the fear that others might be disgusted by them.

### Internalised stigma experiences

#### Self-disgust

The perspective that people with CL wounds are disgusting was internalised as well. Participants explained that they lived in fear that others might be disgusted by them.

You expect people to disgust you. Even I expected that. So, when I see a person with CL wounds, I feel sad because I know how difficult life was for me. They too will have to live with the expectation of facing disgust. (J19)

The belief that the wound and the person with a wound are unattractive was also internalised. People explained how ugly their own wounds looked:

After the treatment, my wound gets dry and flaky. The flakes will then come off. It's ugly to look at. (J10)

## Discussion

We examined the drivers, facilitators and experiences of CL-associated stigma in rural Sri Lanka and, on the basis of that research, we present a conceptual framework of CL-associated stigma (Figure 2). We propose the use of this framework in two ways: as an analysis framework for quantitative and qualitative data on CL-associated stigma and as a structure for the development of stigma-reducing interventions.

Our findings indicate that several factors contribute to stigma, including fear, misconceptions about the disease, the lengthy treatment, the notion that wounds are a disfigurement and blame, which are known drivers of stigma.^11^ Facilitators that can reduce stigma formation include awareness about the curability and non-contagious nature of CL, while facilitators, such as the nature of the social interactions of rural communities, can enhance stigma formation.^12^ Proper awareness can counteract the impact of the cultural context where gathering and gossiping are prevalent.^32^

Existing literature emphasises the need for studying the stigma associated with neglected diseases.^33^ In other stigmatising health conditions, such as mental health^34^ and human immunodeficiency syndrome,^35^ stigma is well understood. Understanding the various forms and consequences of stigma related to CL in specific cultural settings is essential for developing effective public health interventions, as stigma can discourage individuals from seeking healthcare and adhering to treatment.^36^

The framework we present illustrates the experiences of enacted, felt and internalised stigma among people with CL (Figure 2). The potential enacted stigma implications identified in this study, such as disgust directed towards CL patients,^37^ devaluation of body image^37–39^ and mockery^40,41^ align with global findings. Felt stigma experiences of people with CL, including experiencing and anticipating expressions of disgust,^42^ feeling blamed,^43^ stigmatising behaviour by healthcare workers^43^ and marginalisation,^40,42,43^ and internalised stigma experiences such as individuals internalising the belief that their wounds and themselves are unattractive and disgusting^38,43^ were reported in other parts of the world as well. Similar stigma experiences are also found in other skin diseases such as leprosy^44^ and psoriasis.^45^

Literature regarding stereotypes contributing to CL-associated stigma is scarce.^13^ Our study reveals that regular clinic visits for treatment for a prolonged time can lead to the stereotyping of individuals as ‘patients,’ further contributing to stigmatising attitudes. This separation of individuals as ‘patients’ implies weakness and raises concerns about their productivity and meaningful contribution to society, ultimately contributing to the perpetuation of stigma.^12^ Future studies should explore other potential stereotypes at the local and global levels.

Although public stigma related to CL is not currently well-established in the communities studied, the identified potential drivers in this study could contribute to its emergence. In Suriname, it has been observed that CL is not stigmatised, and scholars suggest that this could be attributed to the rarity of facial disfigurements caused by CL in that context.^46^ Similarly, in Sri Lanka, we argue that the low incidence rates and rarity of big wounds associated with CL may be reasons for the absence of a well-established public stigma.^22^ Our ethnographic data offer insights into the absence of public stigma, revealing that in rural areas, people typically pay less attention to minor wounds on body parts like legs due to their common occurrence during farming activities. However, urban areas may exhibit different dynamics, which need to be further investigated.

Ideally, interventions to address stigma should target the identified drivers and facilitators to disrupt stigma formation.^12^ As delineated in the results section, when public stigma is not associated with a disease and individuals are unaware of its stigmatisation, efforts to assure that the disease is not stigmatising may inadvertently provoke the opposite response initially intended. Consequently, interventions should be developed to address the underlying drivers and facilitators of stigma rather than inadvertently inciting public stigma. Our study presents a unique opportunity for the development of public health interventions that address stigma at its early stages and prevent its establishment.

We would like to highlight some limitations of our study. Including people with CL who have completed treatment introduces the possibility of recall bias, potentially leading to the underreporting of nuanced experiences that may have faded from memory over time. Social desirability bias is another potential limitation in this study. Through ethnographic research and increased familiarity, efforts have been made to mitigate its influence.

To further expand our understanding, it is necessary to conduct similar research in urban areas of Sri Lanka and implement studies to quantify and assess the extent of stigma experienced by individuals affected by CL.

## Conclusions

In conclusion, we present a conceptual framework on CL-associated stigma illustrating various drivers, facilitators and self-stigma experiences. Notably, there is a lack of established public stigma associated with CL in rural Sri Lanka. This framework should be used in future research and as a structure to develop stigma-reducing public health interventions. Efforts to reduce self-stigma in Sri Lanka and prevent public stigma formation should focus on dispelling misconceptions, increasing awareness and promoting support for those affected by CL.

## Supplementary Material

ihae021_Supplemental_File

## Data Availability

The datasets used and/or analysed during the current study are available from the corresponding author upon reasonable request.
