# Supplementary material for: Stigma associated with cutaneous leishmaniasis in rural Sri Lanka: development of a conceptual framework
Source: Int Health. 2024 Mar 15;16(5):553–61. doi: 10.1093/inthealth/ihae021 (PMC11375585; doi:10.1093/inthealth/ihae021)
Supplement: ihae021_Supplemental_File [file ihae021_supplemental_file.docx]

| ID | Sex | Age  (Years) | Occupation |
| --- | --- | --- | --- |
| D01* | Female | 45 | Daily wage labourer |
| D02 | Female | 31 | Daily wage labourer |
| D03 | Female | 27 | Daily wage labourer |
| D04 | Female | 50 | Kindergarten teacher |
| D05 | Female | 55 | Self-employed |
| D06 | Male | 55 | Retired army officer |
| D07 | Male | 24 | Daily wage labourer/Traditional dancer |
| D08** | Male | 50 | Government official |
| D09 | Female | 24 | Housewife |
| D10 | Female | 21 | Student |
| D11 | Male | 28 | Development officer |
| D12 | Male | 63 | Retired school teacher |
| D13** | Female | 43 | Housewife |
| D14 | Male | 71 | Farmer |
| D15 | Female | 28 | Development officer |
| D16 | Female | 71 | Housewife |
| D17 | Female | 66 | Traditional healer |
| D18 | Female | 39 | Economic development officer |
| D19 | Male | 48 | Monk |
| D20 | Male | 50 | School teacher |
| D21 | Female | 50 | Retired Army officer |
| D22 | Male | 70 | Farmer |
| D23 | Male | 65 | Famer |
| D24 | Female | 19 | Student |
| D25 | Male | 54 | Monk |
| D26 | Female | 54 | Famer |
| D27 | Male | 51 | Retired army officer |
| D28 | Female | 52 | Farmer |
| D29* | Female | 27 | Management trainee |
| D30 | Female | 26 | Student |

Details of the auto-ethnographic diary study participants

*The diary was not returned

**The follow-up diary interviews were not complete

Socio-demographic and CL lesion characteristics of PERJ participants

| ID | Sex | Age (Years) | Occupation | Location of the lesion(s) | Nature of the lesion |
| --- | --- | --- | --- | --- | --- |
| J01 | Female | 55 | Farmer | Leg | Ulcerated wound |
| J02 | Female | 27 | Daily wage labourer | Leg | Papule |
| J03* | Male | 24 | Helper in a bakery | Hand | N/A |
| J04 | Male | 74 | Retired bank worker | Leg | Wound |
| J05 | Male | 43 | Salesman | Leg | Wound |
| J06 | Female | 31 | Daily wage labourer | Leg | Macule |
| J07 | Female | 32 | Self-employed | Below eye | Papule |
| J08 | Male | 26 | Family business | Ear | Nodule |
| J09 | Male | 36 | Masonry | Hand | Papule |
| J10 | Female | 41 | Unemployed | Leg | Small wound |
| J11* | Male | 71 | Farmer | Hand | N/A |
| J12 | Male | 45 | Farmer/Photographer | Abdomen | Papule |
| J13* | Female | 75 | Unemployed | Hand, Leg | Not done |
| J14 | Male | 56 | Farmer | Leg | Wound |
| J15 | Female | 54 | Unemployed | Finger | Ulcerated wound |
| J16 | Male | 39 | Driver | Hand | Wound |
| J17 | Female | 66 | Unemployed | Leg | Wound |
| J18 | Male | 73 | Unemployed | Hand | Nodule |
| J19 | Male | 44 | Carpenter/Farmer | Hand, Leg | Wound |
| J20 | Male | 32 | Management Assistant | Forehead | Papule |
| J21 | Male | 53 | Daily wage labourer | Ear | Papule |
| J22* | Female | 18 | Unemployed | Leg | N/A |
| J23 | Male | 61 | Farmer | Hand | Wound |
| J24 | Female | 72 | Unemployed | Leg | Wound |
| J25 | Male | 65 | Farmer | Hand | Wound |
| J26* | Male | 32 | Management Assistant | Hand | N/A |
| J27 | Female | 61 | Farmer | Nose | Nodule |
| J28 | Male | 45 | Farmer | Below eye, Back, Eye | Wound |
| J29 | Male | 50 | Retired army officer | Leg | Wound |
| J30 | Female | 47 | School teacher | Leg | Wound |

*PERJ interview was not conducted; N/A
